# Supplementary material for: Pyruvate Dehydrogenase Kinase 1 inhibition mediated oxidative phosphorylation enhancement in cartilage promotes osteoarthritis progression
Source: BMC Musculoskelet Disord. 2023 Jul 20;24:597. doi: 10.1186/s12891-023-06585-6 (PMC10357736; doi:10.1186/s12891-023-06585-6)
Supplement: Supplementary file 1 — Additional file 1. [file 12891_2023_6585_MOESM1_ESM.zip › MF.Gsea.1653627316242/GOMF_KINASE_INHIBITOR_ACTIVITY.html]

Details for gene set GOMF\_KINASE\_INHIBITOR\_ACTIVITY[GSEA]

|  || Dataset | 55457\_collapsed\_to\_symbols.cls.cls #OA\_versus\_normal.cls.cls #OA\_versus\_normal\_repos |
| Phenotype | cls.cls#OA\_versus\_normal\_repos |
| Upregulated in class | normal |
| GeneSet | GOMF\_KINASE\_INHIBITOR\_ACTIVITY |
| Enrichment Score (ES) | -0.38444057 |
| Normalized Enrichment Score (NES) | -1.5146419 |
| Nominal p-value | 0.023217248 |
| FDR q-value | 0.17557497 |
| FWER p-Value | 1.0 |
Table: GSEA Results Summary

  

Fig 1: Enrichment plot: GOMF\_KINASE\_INHIBITOR\_ACTIVITY      
 Profile of the Running ES Score & Positions of GeneSet Members on the Rank Ordered List

  

| SYMBOL | TITLE | RANK IN GENE LIST | RANK METRIC SCORE | RUNNING ES | CORE ENRICHMENT || 1 | NCK1 | NCK adaptor protein 1 [Source:HGNC Symbol;Acc:HGNC:7664] | 222 | 0.804 | 0.0197 | No |
| 2 | CIB1 | calcium and integrin binding 1 [Source:HGNC Symbol;Acc:HGNC:16920] | 436 | 0.691 | 0.0350 | No |
| 3 | CDKN2A | cyclin dependent kinase inhibitor 2A [Source:HGNC Symbol;Acc:HGNC:1787] | 799 | 0.570 | 0.0330 | No |
| 4 | GMFB | glia maturation factor beta [Source:HGNC Symbol;Acc:HGNC:4373] | 867 | 0.551 | 0.0532 | No |
| 5 | PKIA | cAMP-dependent protein kinase inhibitor alpha [Source:HGNC Symbol;Acc:HGNC:9017] | 1598 | 0.414 | 0.0154 | No |
| 6 | SNCA | synuclein alpha [Source:HGNC Symbol;Acc:HGNC:11138] | 1800 | 0.384 | 0.0175 | No |
| 7 | QARS1 | glutaminyl-tRNA synthetase 1 [Source:HGNC Symbol;Acc:HGNC:9751] | 1913 | 0.371 | 0.0258 | No |
| 8 | HSPB1 | heat shock protein family B (small) member 1 [Source:HGNC Symbol;Acc:HGNC:5246] | 2105 | 0.349 | 0.0271 | No |
| 9 | TRIB2 | tribbles pseudokinase 2 [Source:HGNC Symbol;Acc:HGNC:30809] | 2332 | 0.323 | 0.0243 | No |
| 10 | MBIP | MAP3K12 binding inhibitory protein 1 [Source:HGNC Symbol;Acc:HGNC:20427] | 2626 | 0.287 | 0.0147 | No |
| 11 | RHOH | ras homolog family member H [Source:HGNC Symbol;Acc:HGNC:686] | 2680 | 0.282 | 0.0236 | No |
| 12 | CDKN1B | cyclin dependent kinase inhibitor 1B [Source:HGNC Symbol;Acc:HGNC:1785] | 2984 | 0.251 | 0.0116 | No |
| 13 | DUS2 | dihydrouridine synthase 2 [Source:HGNC Symbol;Acc:HGNC:26014] | 3182 | 0.233 | 0.0070 | No |
| 14 | CASP3 | caspase 3 [Source:HGNC Symbol;Acc:HGNC:1504] | 3202 | 0.230 | 0.0161 | No |
| 15 | PKIG | cAMP-dependent protein kinase inhibitor gamma [Source:HGNC Symbol;Acc:HGNC:9019] | 3455 | 0.206 | 0.0060 | No |
| 16 | PARVA | parvin alpha [Source:HGNC Symbol;Acc:HGNC:14652] | 3499 | 0.202 | 0.0119 | No |
| 17 | LILRB4 | leukocyte immunoglobulin like receptor B4 [Source:HGNC Symbol;Acc:HGNC:6608] | 3949 | 0.160 | -0.0156 | No |
| 18 | CDKN2C | cyclin dependent kinase inhibitor 2C [Source:HGNC Symbol;Acc:HGNC:1789] | 4016 | 0.155 | -0.0137 | No |
| 19 | GMFG | glia maturation factor gamma [Source:HGNC Symbol;Acc:HGNC:4374] | 4196 | 0.138 | -0.0212 | No |
| 20 | CDKN1C | cyclin dependent kinase inhibitor 1C [Source:HGNC Symbol;Acc:HGNC:1786] | 4233 | 0.134 | -0.0179 | No |
| 21 | PRKAG2 | protein kinase AMP-activated non-catalytic subunit gamma 2 [Source:HGNC Symbol;Acc:HGNC:9386] | 4362 | 0.123 | -0.0222 | No |
| 22 | TAOK3 | TAO kinase 3 [Source:HGNC Symbol;Acc:HGNC:18133] | 4633 | 0.101 | -0.0385 | No |
| 23 | KAT2B | lysine acetyltransferase 2B [Source:HGNC Symbol;Acc:HGNC:8638] | 4867 | 0.082 | -0.0529 | No |
| 24 | SPRED2 | sprouty related EVH1 domain containing 2 [Source:HGNC Symbol;Acc:HGNC:17722] | 5179 | 0.058 | -0.0744 | No |
| 25 | NCAPG2 | non-SMC condensin II complex subunit G2 [Source:HGNC Symbol;Acc:HGNC:21904] | 5700 | 0.016 | -0.1142 | No |
| 26 | PRKAR1B | protein kinase cAMP-dependent type I regulatory subunit beta [Source:HGNC Symbol;Acc:HGNC:9390] | 5806 | 0.008 | -0.1220 | No |
| 27 | MACROH2A1 | macroH2A.1 histone [Source:HGNC Symbol;Acc:HGNC:4740] | 5953 | -0.002 | -0.1333 | No |
| 28 | RACK1 | receptor for activated C kinase 1 [Source:HGNC Symbol;Acc:HGNC:4399] | 5983 | -0.003 | -0.1354 | No |
| 29 | PRKAR2B | protein kinase cAMP-dependent type II regulatory subunit beta [Source:HGNC Symbol;Acc:HGNC:9392] | 7034 | -0.082 | -0.2134 | No |
| 30 | AHSG | alpha 2-HS glycoprotein [Source:HGNC Symbol;Acc:HGNC:349] | 7041 | -0.083 | -0.2100 | No |
| 31 | PRKRIP1 | PRKR interacting protein 1 [Source:HGNC Symbol;Acc:HGNC:21894] | 7282 | -0.101 | -0.2241 | No |
| 32 | CEP43 | centrosomal protein 43 [Source:HGNC Symbol;Acc:HGNC:17012] | 8016 | -0.156 | -0.2739 | No |
| 33 | SFN | stratifin [Source:HGNC Symbol;Acc:HGNC:10773] | 8118 | -0.164 | -0.2742 | No |
| 34 | HEXIM1 | HEXIM P-TEFb complex subunit 1 [Source:HGNC Symbol;Acc:HGNC:24953] | 8293 | -0.178 | -0.2796 | No |
| 35 | CAMK2N1 | calcium/calmodulin dependent protein kinase II inhibitor 1 [Source:HGNC Symbol;Acc:HGNC:24190] | 8889 | -0.224 | -0.3156 | No |
| 36 | CIT | citron rho-interacting serine/threonine kinase [Source:HGNC Symbol;Acc:HGNC:1985] | 9356 | -0.260 | -0.3399 | No |
| 37 | SPRY2 | sprouty RTK signaling antagonist 2 [Source:HGNC Symbol;Acc:HGNC:11270] | 9531 | -0.277 | -0.3408 | No |
| 38 | PRKAR1A | protein kinase cAMP-dependent type I regulatory subunit alpha [Source:HGNC Symbol;Acc:HGNC:9388] | 10093 | -0.330 | -0.3692 | Yes |
| 39 | WARS1 | tryptophanyl-tRNA synthetase 1 [Source:HGNC Symbol;Acc:HGNC:12729] | 10289 | -0.350 | -0.3683 | Yes |
| 40 | CDKN2B | cyclin dependent kinase inhibitor 2B [Source:HGNC Symbol;Acc:HGNC:1788] | 10324 | -0.353 | -0.3547 | Yes |
| 41 | MAPK8IP1 | mitogen-activated protein kinase 8 interacting protein 1 [Source:HGNC Symbol;Acc:HGNC:6882] | 10389 | -0.360 | -0.3431 | Yes |
| 42 | HYAL2 | hyaluronidase 2 [Source:HGNC Symbol;Acc:HGNC:5321] | 10420 | -0.363 | -0.3287 | Yes |
| 43 | PRKAR2A | protein kinase cAMP-dependent type II regulatory subunit alpha [Source:HGNC Symbol;Acc:HGNC:9391] | 10664 | -0.389 | -0.3297 | Yes |
| 44 | WNK1 | WNK lysine deficient protein kinase 1 [Source:HGNC Symbol;Acc:HGNC:14540] | 10777 | -0.403 | -0.3198 | Yes |
| 45 | TRIB3 | tribbles pseudokinase 3 [Source:HGNC Symbol;Acc:HGNC:16228] | 10839 | -0.409 | -0.3057 | Yes |
| 46 | CHP1 | calcineurin like EF-hand protein 1 [Source:HGNC Symbol;Acc:HGNC:17433] | 10940 | -0.423 | -0.2940 | Yes |
| 47 | YWHAB | tyrosine 3-monooxygenase/tryptophan 5-monooxygenase activation protein beta [Source:HGNC Symbol;Acc:HGNC:12849] | 11488 | -0.500 | -0.3136 | Yes |
| 48 | SH3BP5 | SH3 domain binding protein 5 [Source:HGNC Symbol;Acc:HGNC:10827] | 11545 | -0.506 | -0.2946 | Yes |
| 49 | TESC | tescalcin [Source:HGNC Symbol;Acc:HGNC:26065] | 11793 | -0.548 | -0.2886 | Yes |
| 50 | TRIB1 | tribbles pseudokinase 1 [Source:HGNC Symbol;Acc:HGNC:16891] | 11924 | -0.577 | -0.2722 | Yes |
| 51 | IBTK | inhibitor of Bruton tyrosine kinase [Source:HGNC Symbol;Acc:HGNC:17853] | 11966 | -0.586 | -0.2483 | Yes |
| 52 | INKA2 | inka box actin regulator 2 [Source:HGNC Symbol;Acc:HGNC:28045] | 11992 | -0.592 | -0.2230 | Yes |
| 53 | LRP6 | LDL receptor related protein 6 [Source:HGNC Symbol;Acc:HGNC:6698] | 12051 | -0.607 | -0.1996 | Yes |
| 54 | CDKN2D | cyclin dependent kinase inhibitor 2D [Source:HGNC Symbol;Acc:HGNC:1790] | 12296 | -0.676 | -0.1875 | Yes |
| 55 | SPRY4 | sprouty RTK signaling antagonist 4 [Source:HGNC Symbol;Acc:HGNC:15533] | 12441 | -0.721 | -0.1655 | Yes |
| 56 | SOCS3 | suppressor of cytokine signaling 3 [Source:HGNC Symbol;Acc:HGNC:19391] | 12453 | -0.725 | -0.1329 | Yes |
| 57 | NPM1 | nucleophosmin 1 [Source:HGNC Symbol;Acc:HGNC:7910] | 12530 | -0.762 | -0.1037 | Yes |
| 58 | SOCS1 | suppressor of cytokine signaling 1 [Source:HGNC Symbol;Acc:HGNC:19383] | 12664 | -0.836 | -0.0756 | Yes |
| 59 | CDKN1A | cyclin dependent kinase inhibitor 1A [Source:HGNC Symbol;Acc:HGNC:1784] | 12727 | -0.890 | -0.0394 | Yes |
| 60 | DNAJC3 | DnaJ heat shock protein family (Hsp40) member C3 [Source:HGNC Symbol;Acc:HGNC:9439] | 12876 | -1.146 | 0.0019 | Yes |
Table: GSEA details [plain text format]

  

Fig 2: GOMF\_KINASE\_INHIBITOR\_ACTIVITY      
 Blue-Pink O' Gram in the Space of the Analyzed GeneSet

  

Fig 3: GOMF\_KINASE\_INHIBITOR\_ACTIVITY: Random ES distribution      
 Gene set null distribution of ES for **GOMF\_KINASE\_INHIBITOR\_ACTIVITY**
